# Screening of Anti-Inflammatory Components of Qin Jin Hua Tan Tang by a Multivariate Statistical Analysis Approach for Spectrum-Effect Relationships

**DOI:** 10.1155/2021/6348979

**Published:** 2021-08-13

**Authors:** Feipeng Duan, Yisheng Li, Meizhen Zhao, Tianyong Hu, Xinquan Pan, Yue Feng, Fang Ma, Shuqi Qiu, Yiqing Zheng

**Affiliations:** ^1^Department of Otolaryngology, Longgang E. N. T Hospital and Shenzhen Key Laboratory of E. N. T, Institute of E. N. T, Shenzhen 518172, China; ^2^Institute of Otolaryngology, Institute of Hearing and Speech of Sun Yat-sen University, Sun Yat-sen Memorial Hospital, Sun Yat-sen University, Guangzhou 510520, China

## Abstract

Qing Jin Hua Tan Tang (QJHTT) exerts therapeutic effects in patients with chronic obstructive pulmonary disease (COPD) by alleviating inflammation. However, the anti-inflammatory components of QJHTT have not yet been reported. Our study aimed to screen the active anti-inflammatory components of QJHTT using a multivariate statistical analysis approach for spectrum-effect relationships. Different polar fractions of QJHTT were prepared using ethanol, ethyl acetate, and *n*-butanol to analyze the phytochemical components. Phytochemical fingerprints were generated using ultrahigh-performance liquid chromatography. In total, 24 peaks were observed in ten batches of QJHTT extracts. The anti-inflammatory activity was evaluated using a xylene-induced ear-swelling mouse model. Additionally, the spectrum-effect relationship between the relative areas of the 24 peaks and pharmacological activity was investigated using multivariate statistical analysis. The potential anti-inflammatory ingredients obtained from the screening (multivariate statistical analysis) will be validated for their anti-inflammatory effects and mechanisms utilizing a lipopolysaccharide-induced macrophage inflammation model. QJHTT ethanol extract 1 exhibited good anti-inflammatory activity. Peaks 11, 12, 13, 14, and 16, which were closely correlated with anti-inflammatory activity, were identified as meranzin, baicalin, baicalein, chrysin-7-O-*β*-D-glucuronide, and wogonoside, respectively. The anti-inflammatory activities of meranzin, baicalin, baicalein, and wogonoside were verified *in vitro*. These four bioactive components significantly inhibited the secretion of inflammatory factors in the lipopolysaccharide-stimulated macrophage cell line. This research successfully screened the QJHTT anti-inflammatory active ingredient group. Meranzin, baicalin, baicalein, chrysin-7-O-*β*-D-glucuronide, and wogonoside were predicted to be the anti-inflammatory active ingredient groups of QJHTT.

## 1. Introduction

Qing Jin Hua Tan Tang (QJHTT), a traditional Chinese medicine (TCM) prescription (CMP), is widely used to treat phlegm heat cough syndrome, which is characterized by cough, pulmonary carbuncle, and pulmonary distension. In modern medicine, symptoms of phlegm heat cough syndrome correspond to those of chronic obstructive pulmonary disease (COPD) [1]. QJHTT is composed of the following 11 herbs: Scutellariae Radix, Gardeniae Fructus, Platycodonis Radix, Ophiopogonis Radix, Mori Cortex, Anemarrhenae Rhizoma, Trichosanthis Semen, Citri Grandis Exocarpium, Fritillariae Cirrhosae Bulbus, Poria, and Glycyrrhizae Radix et Rhizoma. Pharmacological studies have demonstrated the anti-inflammatory effects of QJHTT in patients with COPD can significantly alleviate acute airway inflammation [2]. Flavonoids in Scutellariae Radix exhibit anti-inflammatory activity in lipopolysaccharide- (LPS-) induced RAW 264.7 cells (macrophages) [3]. The ethanolic extract of Platycodonis Radix mitigates LPS-induced inflammation in pulmonary epithelial cells (A549 cells) [4, 5]. Isosteroid alkaloids isolated from Fritillariae Cirrhosae Bulbus exerted anti-inflammatory effects in RAW 264.7 cells by regulating MAPK phosphorylation [6]. In 2018, QJHTT was selected for the first batch of the “Classical Chinese Medicine Classical Directory,” as it exhibited potent pharmacological activities against COPD. The anti-inflammatory activity of QJHTT has been previously reported. However, the components contributing to the anti-inflammatory activity of QJHTT have not been elucidated.

Activation of the inflammatory response can contribute to the progression of chronic diseases, such as COPD and metabolic diseases [7]. COPD is associated with local pulmonary inflammation and systemic inflammation, which are mainly localized in the peripheral airways and lung parenchyma [8]. Inflammation-induced fibrosis and peripheral airway collapse lead to airway obstruction, which contributes to the development of cough and asthma [9]. The major treatment for COPD is the administration of anti-inflammatory agents. NF-*κ*B, which is a vital regulator of the inflammatory response, modulates the transcription of IL-6 and TNF-*α* [10]. QJHTT is a classical TCM for the treatment of COPD; however, the lack of clarity regarding the active ingredients significantly limits the clinical application of QJHTT.

The traditionally used dosage form is a decoction characterized by large doses of medicine and inconvenience in carrying. Therefore, screening of the active group of QJHTT can overcome these disadvantages and be helpful for secondary development. In contrast to those in single-herbal medicines, bioactive components in multiherbal formulations are identified based on the correlation between different original single-herbal chemical fingerprints and efficacy indices. QJHTT comprises 11 traditional Chinese herbal medicines. Hence, the different origins of 11 herbal correlation analyses would be laborious work to identify the anti-inflammatory ingredients of QJHTT. Herein, we constructed fingerprint profiles of the different polar sites of QJHTT extracts, evaluated their anti-inflammatory activity, and screened their active anti-inflammatory ingredients using a spectrum-effect relationship analysis. To date, spectrum-effect relationship analysis has been successfully used to screen the active ingredients in single-herbal medicine and CMP [11]. The spectrum-effect relationship between chemical fingerprints of CMP and the bioactivity index was examined using multivariate statistical analysis [12, 13]. Modernization of CMP analysis has been achieved through the application of multivariate statistical models, such as gray relational analysis (GRA) and partial least squares regression (PLSR), to analyze the spectrum-effect relationship [14]. In addition, redundancy analysis (RDA) was first applied to the spectrum-effect relationship, which maps the ingredients and bioactivity index onto the same two-dimensional ordination diagram, allowing for a visual representation of the correlation between ingredients and the bioactivity index. Systematic screening of QJHTT anti-inflammatory active ingredients was conducted using a combination of three statistical analysis methods.

In this study, the polar components of QJHTT were extracted using different solvents. The anti-inflammatory components of QJHTT could be rapidly and efficiently screened based on the correlation between the concentration of different polar components and their anti-inflammatory activities. Furthermore, the potential anti-inflammatory components were validated using an *in vitro* screening test.

## 2. Materials and Methods

### 2.1. Plant Materials and Reagents

All herbal materials of QJHTT were purchased from Kangmei Pharmaceutical Co., Ltd. (Pulin, China). The sources of the herbal materials used in QJHTT are shown in Table 1. The quality of herbal materials complied with the National 2015 Pharmacopeia standards. The raw materials were identified by Yisheng Li (Deputy Director of TCM, Pharmacy of Traditional Chinese Medicine, Longgang ENT Hospital). The medicinal materials were stored at the Laboratory of Research on New Drugs of Traditional Chinese Medicine, Longgang ENT Hospital. Methanol (Optima® LC/MS grade) was purchased from Fisher Scientific (Waltham, MA, USA). Ethyl acetate, xylene, and *n*-butyl alcohol, all of analytical grade, were obtained from Sinopharm Chemical Reagent Co., Ltd. (Beijing, China). LPS was purchased from Sigma-Aldrich (St. Louis, MO, USA). The TNF-*α* enzyme-linked immunosorbent assay (ELISA) kit was purchased from Cayman Chemical (Michigan, USA). IL-4 (mouse) and IL-6 (mouse) ELISA kits were purchased from Enzo Life Sciences (New York, USA). Dulbecco's modified Eagle's medium (DMEM), fetal bovine serum (FBS), and penicillin/streptomycin were purchased from Thermo Fisher Scientific Inc. (Waltham, USA).

### 2.2. Animals

Male Kunming mice (4–6 weeks with an average body weight of 25–30 g) were obtained from the Center of Laboratory Animal Science of Guangdong (quality exequatur number: SCXK 20180002). The animals, which were housed under standard temperature, humidity, and light conditions, had free access to water. The mice were allowed to fast for 12 h prior to the experiments. All procedures were approved by the Institutional Animal Use Committee (Ethics Committee of ZSSOM on Laboratory Animal Care, No. 2020–0112).

### 2.3. Sample Preparation

The procedure for the preparation of ten polar extracts from QJHTT is described in Supplementary Materials 1. Water extracts were prepared from QJHTT (WE; WE-1, high-dose; WE-2, low-dose). Ethyl acetate (EAWE; EAWE-1, high-dose; EAWE-2, low-dose) and *n*-butyl alcohol (NBWE; NBWE-1, high-dose; NBWE-2, low-dose) fractions were prepared using WE. Alcohol extracts (AE; AE-1, high-dose; AE-2, low-dose) were prepared from QJHTT. Ethyl acetate fractions were prepared from AE (EAAE; EAAE-1, high-dose; EAAE-2, low-dose). The extract (10 and 5 mg for the high-dose and low-dose extracts, respectively) was dissolved in 10 mL of 50% methanol with distilled water. The mixture was filtered through a 0.45 *μ*m membrane, and 2 *µ*L was injected into an ultrahigh-performance liquid chromatography (UHPLC) system to obtain the chromatogram of QJHTT.

### 2.4. Chromatography Conditions

The phytochemical analysis of QJHTT was performed using an Agilent UHPLC system coupled to a quadrupole time-of-flight (Q-TOF) tandem mass spectrometer (MS/MS) (Agilent Technology, USA) with an electrospray ionization (ESI) interface.

The chromatographic conditions were as follows: column, C18 reversed-phase column ACCQ-TAGTMULTRA (2.1 mm × 100 mm; 1.7 *μ*m; Agilent Technologies); mobile phase, 0.1% formic acid in water (eluent A) and methanol (eluent B); flow rate, 0.3 mL/min; column temperature, 30°C; injection volume, 2 *μ*L. The gradient program was as follows: 0 min, 7% eluent B; 2 min, 7% eluent B; 8 min, 20% eluent B; 12 min, 25% eluent B; 20 min, 35% eluent B; 30 min, 65% eluent B; 35 min, 80% eluent B; 40 min, 90% eluent B; 45 min, 90% eluent B; 46 min, 7% eluent B. The chromatogram was captured at a wavelength of 360 nm during the experiment. The results of the optimization of the detection wavelength are presented in Figure S1.

Mass spectrometry was performed in both positive and negative ion modes with the following parameters: gas temperature, 300°C; drying gas flow rate, 10 L/min; sheath gas temperature, 365°C; sheath gas flow rate, 12 L/min; nebulizer gas pressure, 45 psi. Potential active compounds were identified using a Q-TOF analyzer. The data were collected and processed using Mass Hunter software (version B.07.00).

### 2.5. Methodological Validation

EAWE-1 was repetitively injected with 20 *µ*L five times to confirm the precision of the instruments. The RSD values of the major chromatograph peak area were all less than 2.0%, and the RSD values of the relative retention times of the major chromatograph peaks were all below 2.0%, suggesting that the instrument had good precision. EAWE-1 was analyzed at 0, 2, 4, 8, 12, and 24 h to evaluate the stability of the sample at room temperature. The RSD values of the major chromatograph peak area at different times were all less than 5.0%, suggesting that the sample was stable within 24 h. Five batches of EAWE-1 were prepared following the method described in Section 2.3. The RSD values of the major chromatograph peak area of the five batches of EAWE-1 were all below 5%, suggesting that the method had good repeatability.

### 2.6. Anti-Acute Inflammatory Effect of Different Polar QJHTT Extracts

Mice were randomly divided into the following groups: model, positive control, and extract-treated groups. The mice were administered the target drugs by gavage once a day for seven consecutive days. The treatment regimens for different groups (7 mice/group) were as follows: model group, administered with 0.5% sodium carboxymethylcellulose (CMC-Na) (0.2 mL/25 g bodyweight/day); positive control group, administered with dexamethasone acetate (0.54 mg/kg bodyweight/day); low-dose extract-treated group, administered with extracts at a dose of 200 mg/kg bodyweight/day; high-dose extract-treated group, administered with extracts at a dose of 400 mg/kg bodyweight/day. At 1 h after drug administration, 20 *μ*L xylene was applied to the right ear (but not the left ear) of the mice in all treatment groups for 30 min. The mice were sacrificed by cervical dislocation under ether anesthesia, and two ear punches were collected. The difference in the weight of the left and right ear punches was calculated using the weight difference (WD). The swell inhibition rate (SIR) was calculated using the following formula [15]:

SIR % = (1 − WD of the extract-treated group/WD of the model group) × 100.

### 2.7. Spectrum-Effect Relationship Analysis

#### 2.7.1. GRA

GRA was performed using the Data Processing System (DPS V15.10) to examine the correlation between the compounds and pharmacodynamics. The relative areas of the 24 peaks (*P*1–*P*24) were standardized before performing the GRA. The standardized data were selected as the subsequences, and the SIR levels were defined as the parent subsequence [16]. The resolution coefficient (*ξ*) was 0.5, after calculating the gray relational coefficients and grades for 24 peak areas.

#### 2.7.2. PLSR Analysis

PLSR analysis was performed using Simca-P13.0 (Umetrics, Sweden) to establish the statistical correlation between multiple dependent and independent variables. In this study, 24 chromatographic peaks were used as independent variables, while SIR was used as a dependent variable to generate a regression model. The correlation between the peak areas and SIR was analyzed using the PLSR model [17].

#### 2.7.3. RDA for Screening Anti-Inflammatory Bioactive Components

RDA was performed to further demonstrate the direct correlation between the component peak area and bioactivity. This is a direct gradient analysis method that enables a statistical evaluation of the correlation between one or a set of variables and another set of multivariate data [18, 19]. In this study, RDA was performed using CANOCO 4.5, to analyze the correlation between the 24 peak areas and efficacy indices (anti-inflammatory activity).

### 2.8. UHPLC Analysis and Peak Identification

The chromatograms of 10 samples (WE-1, WE-2, EAWE-1, EAWE-2, NBWE-1, NBWE-2, AE-1, AE-1, EAAE-1, and EAAE-2) were obtained using UHPLC. The mass spectrum peaks in both positive and negative ion modes were identified based on the accurate mass and precursor ions along with the corresponding secondary fragment ion. The bioactive phytochemical components of the 10 samples with potent anti-inflammatory activity were identified. Standard reference controls for the identified components were used for further analysis.

### 2.9. Verification of Anti-Inflammatory Components of QJHTT

#### 2.9.1. Cell Culture

RAW264.7 cells were obtained from the American Type Culture Collection (Manassas, USA; passages 10–20). The cells were cultured in DMEM supplemented with 10% FBS and 1% penicillin/streptomycin solution in a humidified chamber at 5% CO_2_ and 37°C.

#### 2.9.2. Cell Viability Test

RAW264.7 cells were cultured in DMEM supplemented with 10% FBS and 1% penicillin/streptomycin solution in a humidified chamber at 5% CO_2_ and 37°C. The cells were cultured in 96-well plates (5 × 10^4^ cells/well) for 24 h and treated with various concentrations (0, 5, 10, 20, 40, 80, and 160 *µ*M) of meranzin, baicalin, baicalein, or wogonoside for 24 h. Cells in each well were incubated with 20 *μ*L of 3-(4,5-dimethylthiazol-2-yl)-2,5-diphenyltetrazolium bromide (MTT; 5 mg/mL) for 4 h. The formazan crystals were solubilized in dimethyl sulfoxide (DMSO; 100 *μ*L/well). The absorbance of the mixture was measured at 490 nm using a SpectraMax Paradigm Multi-Mode Microplate Reader.

#### 2.9.3. ELISA Test

RAW264.7 cells (5 × 10^4^ cells/well) were cultured in 96-well plates for 12 h and treated with meranzin, baicalin, baicalein, or wogonoside (20 *µ*M) for 2 h. Cells in the model group were treated with an equal volume of basal medium. Next, the cells were stimulated with 1 *µ*g/mL LPS for 24 h. The LPS-induced production of TNF-*α* and IL-6 in the culture supernatant was analyzed using an ELISA kit according to the manufacturer's instructions [20].

#### 2.9.4. Western Blotting

RAW264.7 cells (1 × 10^6^ cells/well) were cultured in six-well plates for 12 h and treated with meranzin, baicalin, baicalein, or wogonoside (20 *µ*M) for 2 h. Cells in the model group were treated with an equal volume of basal medium. Next, the cells were stimulated with 1 *µ*g/mL LPS for 24 h. The cells were washed twice with ice-cold phosphate-buffered saline (pH 7.5) and subjected to nuclear and cytoplasmic protein extraction using a nuclear extraction kit (Beyotime, China). Protein concentration was determined using a bicinchoninic acid protein assay kit (Beyotime, China). Equal amounts (10 *µ*g) of protein were subjected to sodium dodecyl sulfate-polyacrylamide gel electrophoresis on a 10% gel. The resolved proteins were transferred onto polyvinylidene difluoride membranes. The membrane was blocked with 5% bovine serum albumin and incubated with the following antibodies at 4°C overnight: anti-NF-*κ*B (p65) (1 : 1000, Abcam, UK), anti-*β*-actin (1 : 1000, Abcam, UK), anti-IKB-*α* (1 : 1000, Beyotime, China), and anti-Lamin B1 (1 : 1000, Beyotime, China) antibodies. Next, the membrane was washed five times with Tris-buffered saline (TBS, pH 7.5) and incubated with the peroxidase-conjugated secondary antibody (1 : 1000) at room temperature (25°C) for 2 h. The membrane was washed five times with TBST (TBS containing 0.05% Tween-20), and the immunoreactive bands were developed using an enhanced chemiluminescence (ECL) reagent (Beyotime, China). The band density was analyzed using the ChemiDoc^TM^ MP Imaging System (BioRad, USA).

### 2.10. Statistical Analysis

All data are expressed as the mean ± standard deviation. Statistical significance was analyzed by one-way ANOVA and a least significant difference (LSD) test for multiple comparisons. All statistical analyses were performed using SPSS (version 12.0; IBM, USA).

## 3. Results

### 3.1. UHPLC Chromatogram of Different Polar Fractions of QJHTT Extracts

**Figure 1:**
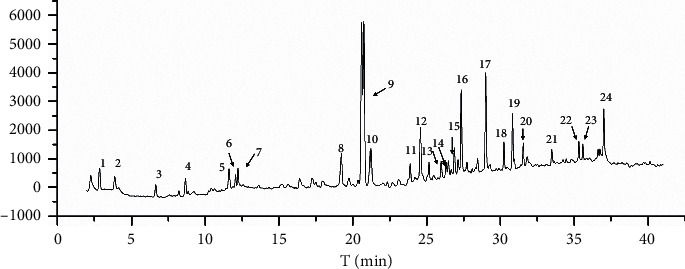
Chromatogram (captured at 360 nm) of NBWE-1 captured using ultrahigh-performance liquid chromatography.

**Figure 2:**
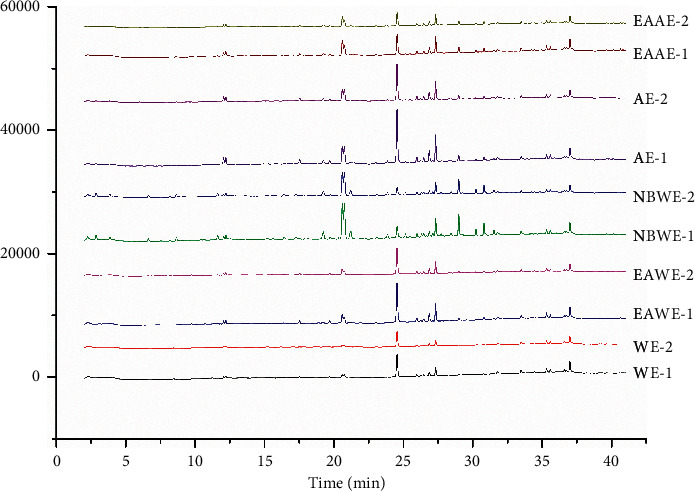
Chromatograms (360 nm) of 10 Qing Jin Hua Tan Tang extracts captured using ultrahigh-performance liquid chromatography.

The UHPLC-UV chromatogram of NBWE-1 is shown in Figure 1. Analysis of the UHPLC-UV chromatograms (360 nm) of the ten QJHTT extracts (Figure 2) revealed variations in the phytochemical components. In total, 24 peaks were identified in all 10 extracts. The relative retention times and areas of the 24 peaks are shown in Table S1.

### 3.2. Effect of QJHTT Extracts on Xylene-Induced Ear Swelling in Mice

The weight of the ear punch (with a diameter of 6 mm) was measured after the administration of QJHTT or dexamethasone acetate following xylene treatment. As shown in Table 2, dexamethasone acetate markedly inhibited xylene-induced swelling. The SIRs of WE-1, WE-2, EAWE-1, EAWE-2, NBWE-1, NBWE-2, AE-1, AE-1, EAAE-1, and EAAE-2 were 33.9, 23.1, 69.3, 55.3, 71.3, 62.5, 74.3, 48.6, 34.8, and 27.6%, respectively. AE-1 exhibited the most potent anti-inflammatory activity.

### 3.3. Analysis of the Spectrum-Effect Relationship

#### 3.3.1. GRA

The correlation coefficient is classified in three grades (>0.8 means strong correlation, 0.7–0.8 means moderate correlation, and <0.7 means low correlation) [21]. As shown in Table 3, the coefficient of correlation of peaks 13, 16, 14, 11, and 12 with the anti-inflammatory activity of QJHTT was higher than 0.7, which indicated a moderate correlation. The coefficient of correlation of peaks 9, 6, 21, 7, 24, 19, 4, 17, 5, 1, 20, 8, 2, 18, 10, 22, and 3 with the anti-inflammatory activity of QJHTT was between 0.6 and 0.7, which indicated a low correlation. These findings indicate that QJHTT contains multiple components that synergistically exert beneficial effects. Peaks 11, 12, 13, 14, and 16 were the top four ranked peaks correlated with anti-inflammatory activity and hence may be the major bioactive components of QJHTT.

#### 3.3.2. PLSR Analysis

**Figure 3:**
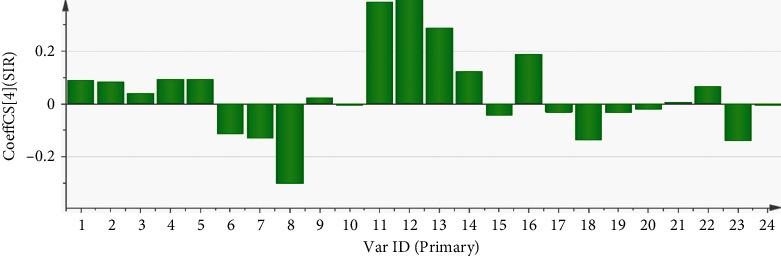
Coefficient of correlation of 24 chromatographic peaks with anti-inflammatory activity.

In the PLSR model analyzing 24 chromatographic peak areas and SIR, the first four principal components explained the variances of the *x* and *y* variables, which were 96.3% and 97.2%, respectively, and the variance of *Q*2 was 0.823. This indicates that the proposed PLSR model has good adaptability and predictability. The regression equation obtained using Simcap 13.0 software was as follows: *Y*_SIR_ = 0.092 × *P*_1_ + 0.085 × *P*_2_ + 0.042 × *P*_3_ + 0.096 × *P*_4_ + 0.094 × *P*_5_ − 0.115 × *P*_6_ − 0.131 × *P*_7_ − 0.302 × *P*_8_ + 0.024 × *P*_9_ − 0.007 × *P*_10_ + 0.387 × *P*_11_ + 0.409 × *P*_12_ + 0.291 × *P*_13_ + 0.124 × *P*_14_ − 0.043 × *P*_15_ + 0.189 × *P*_16_ − 0.032 × *P*_17_ − 0.137 × *P*_18_ − 0.034 × *P*_19_ − 0.022 × *P*_20_ + 0.006 × *P*_21_ + 0.068 × *P*_22_ − 0.14 × *P*_23_ − 0.006 × *P*_24_. The correlation coefficient plot of PLSR analysis is shown in Figure 3 [22].

The magnitude of the correlation coefficient, which indicates the degree of correlation between the phytochemical components and pharmacological activity, is directly proportional to the degree of correlation. A correlation coefficient value greater than zero indicates that the component has a positive regulatory effect on pharmacological activity. Conversely, a correlation coefficient value of less than zero indicates that the component has a negative regulatory effect on the activity. The PLSR model revealed that peaks 11, 12, 13, 14, and 16 positively modulate anti-inflammatory activity. This result is consistent with that of the GRA.

**Figure 4:**
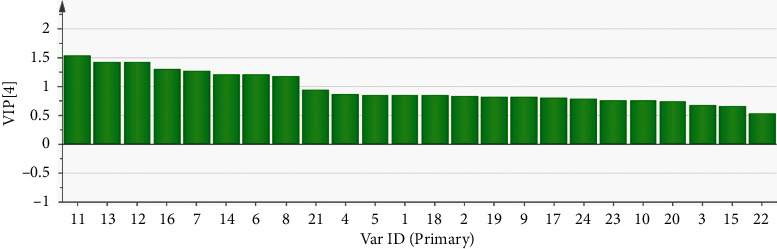
Variable importance in projection scores predicted the anti-inflammatory activity of 24 chromatographic peaks in the Qing Jin Hua Tan Tang extracts.

Variable importance in projection (VIP) scores indicate the contribution of independent variables to the dependent variables [16]. High VIP scores indicated an enhanced contribution of the components to biological activities. The VIP scores of the 24 components are plotted in Figure 4. The predictive abilities of the independent variables were comparatively analyzed. A VIP score greater than 1 indicates that the independent variable has a marked contribution to the dependent variable. Thus, components with a VIP score greater than 1 markedly contribute to the anti-inflammatory activity. As shown in Figure 4, the VIP scores of peaks 11, 13, 12, 16, 7, 14, 6, and 8 were higher than 1. This indicated that these components markedly contributed to the anti-inflammatory activity.

#### 3.3.3. RDA

**Figure 5:**
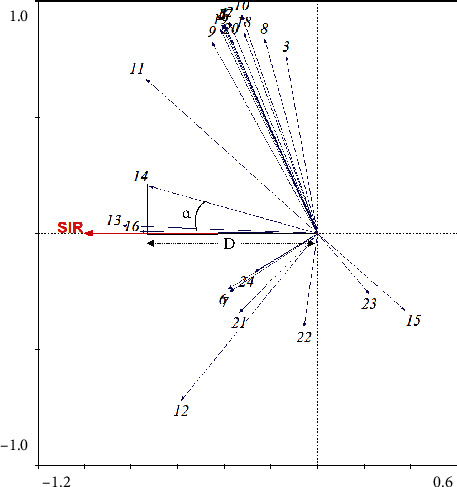
Redundancy analysis of phytochemical components and efficacy index.

In the RDA sample location plot (Figure 5), 24 components and efficacy indices are shown in the same coordinate system. The angle (*α*) between the component arrow and the efficacy index arrow can predict the correlation between the component and efficacy indices. The magnitude of the angle is inversely proportional to the degree of correlation. The correlation is negative when the angle is greater than 90°. Distance (*D*) from the projection point to the start of the efficacy indicator arrow, which is a point projection for each component, can indicate the contribution of the component to the efficacy indices. Peaks 1, 2, 3, 4, 5, 6, 7, 8, 9, 10, 11, 12, 13, 14, 16, 17, 18, 19, 20, 21, 22, and 24 were positively correlated with the anti-inflammatory indicator. The top 10 peaks that contributed to the efficacy index were peaks 16, 13, 14, 11, 24, 6, 7, 21, 12, and 9. Comparative analysis of the GRA, PLSR, and RDA results revealed that peaks 11, 12, 13, 14, and 16 had a marked impact on anti-inflammatory activity.

### 3.4. Identification of Active Components Using UPLC-Q-TOFMS/MS

**Figure 6:**
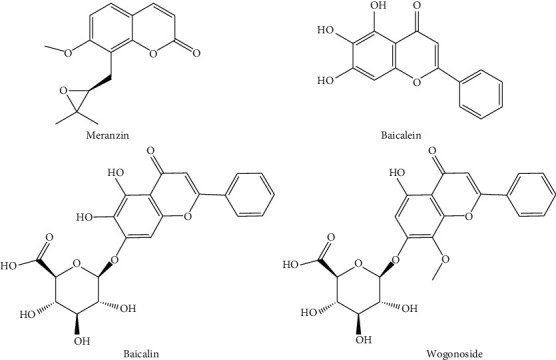
Chemical structures of meranzin, baicalin, baicalein, and wogonoside.

**Figure 7:**
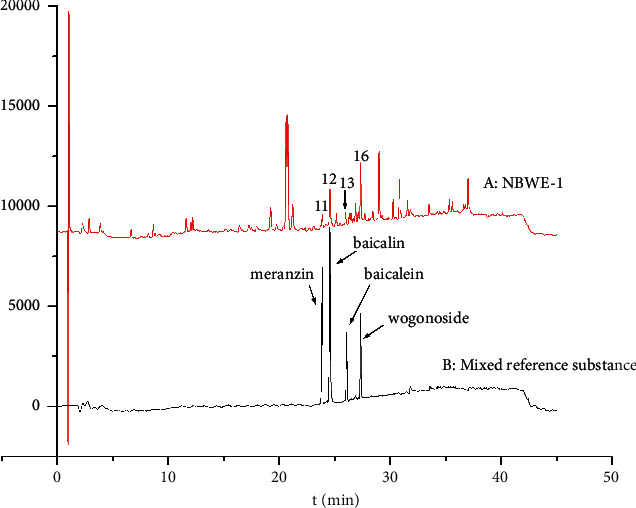
Comparison of chromatograms of Qing Jin Hua Tan Tang extracts and reference compounds.

UPLC-Q-TOF MS/MS was used to quantify the potential active components. Peaks 11, 12, 13, 14, and 16 were identified as meranzin, baicalin, baicalein, chrysin-7-O-beta-D-glucuronide, and wogonoside, respectively. The MS data are listed in Table S2. The product ion spectra of meranzin, baicalin, baicalein, chrysin-7-O-Beta-D-glucuronide, and wogonoside are shown in Figures S2–S6. Standard meranzin, baicalin, baicalein, and wogonoside (Figure 6) were purchased from the National Institutes for Food and Drug Control (chrysin-7-O-beta-D-glucuronide was not commercially available). The retention times for meranzin, baicalin, baicalein, and wogonoside standards were identical to those of *P*11, *P*12, *P*13, and *P*16, respectively (Figure 7). Quantitative results of *P*11, *P*12, *P*13, and *P*16 in 10 extracts are presented in Table S3.

### 3.5. Anti-Inflammatory Activity of Potential Active Components

#### 3.5.1. Cytotoxic Effects of Four Potential Anti-Inflammatory Components against Macrophage Cells

**Figure 8:**
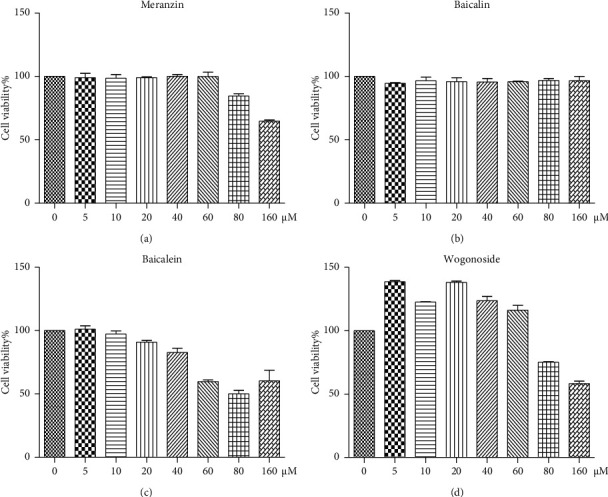
Cytotoxic activities of various concentrations (5, 10, 20, 40, 80, and 160 *µ*M) of meranzin, baicalin, baicalein, and wogonoside against RAW264.7 cells after 24 h of treatment. Results are expressed as mean ± SD from three independent experiments (*n* = 3).

The cytotoxic effects of various concentrations (0, 5, 10, 20, 40, 80, and 160 *µ*M) of meranzin, baicalin, baicalein, and wogonoside against RAW264.7 were evaluated using the MTT assay (Figure 8). The viability of cells treated with 20 *µ*M meranzin, baicalin, baicalein, or wogonoside was more than 90%. Therefore, 20 *µ*M was selected as the experimental concentration for subsequent experiments.

#### 3.5.2. Effects of Four Potential Anti-Inflammatory Components on Proinflammatory Cytokine Production

**Figure 9:**
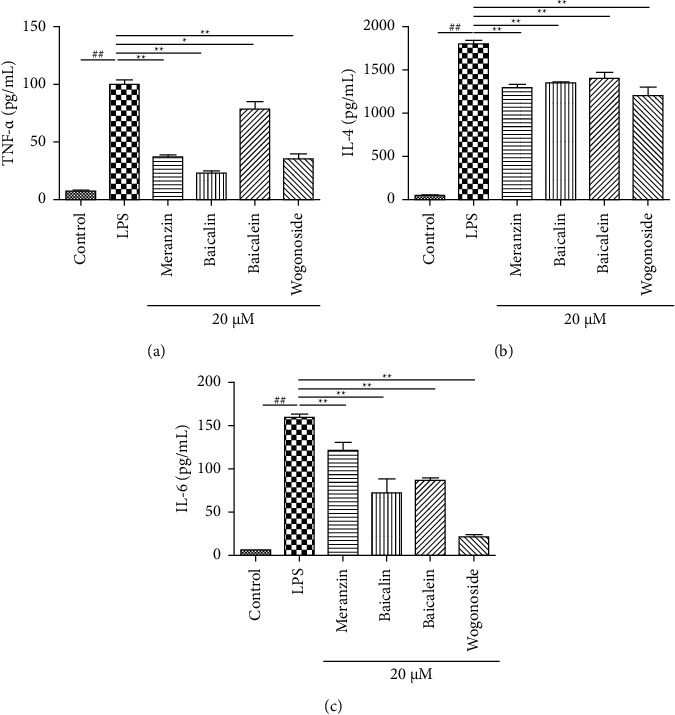
Effects of meranzin, baicalin, baicalein, and wogonoside on the production of TNF-*α*, IL-4, and IL-6 in lipopolysaccharide- (LPS-) stimulated RAW264.7 cells. The model group was treated with a basal medium for 2 h and then stimulated with 1 *µ*g/mL LPS for 24 h. Results are expressed as mean ± SD from three independent experiments (*n* = 3). ##*P* < 0.01, compared with the control group; ^*∗∗*^*P* < 0.01, compared with the LPS-treated group.

TNF-*α*, IL-6, and IL-4 are critical mediators of inflammation and lung injury [23]. The effects of meranzin, baicalin, baicalein, and wogonoside on the LPS-induced secretion of TNF-*α*, IL-6, and IL-4 were examined using ELISA. The levels of TNF-*α*, IL-6, and IL-4 were significantly upregulated in LPS-induced cells (Figure 9). Treatment with meranzin, baicalin, baicalein, and wogonoside significantly mitigated the LPS-induced upregulation of TNF-*α*, IL-6, and IL-4. The highest inhibition of TNF-*α* and IL-6 secretion was observed in the baicalin-treated (Figure 9(a)) and wogonoside-treated cells (Figure 9(c)). Thus, the anti-inflammatory activity of meranzin, baicalin, baicalein, and wogonoside, predicted using spectrum-effect relationship analysis, was validated experimentally.

#### 3.5.3. Four Potential Anti-Inflammatory Components Inhibit the Nuclear Translocation of NF-*κ*B

**Figure 10:**
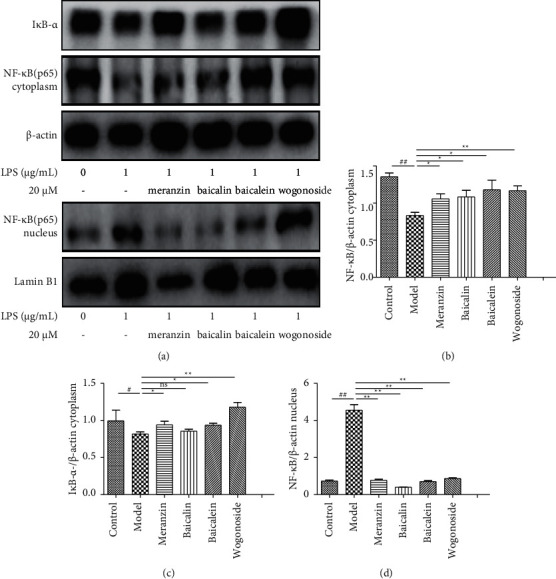
Effects of meranzin, baicalin, baicalein, and wogonoside on the nuclear translocation of NF-*κ*B in the lipopolysaccharide-stimulated RAW264.7 cells. The model group was treated with the basal medium for 2 h and then stimulated with 1 *µ*g/mL LPS for 24 h. Results are expressed as mean ± SD from three independent experiments (*n* = 3).

NF-*κ*B mediates the inflammatory process in COPD by enhancing the transcription of proinflammatory cytokine-encoding genes [24]. In this study, the effects of meranzin, baicalin, baicalein, and wogonoside on the nuclear translocation of NF-*κ*B were examined. As shown in Figures 10(a) and 10(b), treatment with meranzin, baicalin, baicalein, and wogonoside alleviated the LPS-induced downregulation of cytoplasmic levels of NF-*κ*B. All components, except wogonoside, increased the nuclear levels of NF-*κ*B (Figures 10(a) and 10(d)). Furthermore, the level of I*κ*B-*α* in the meranzin-treated, baicalin-treated, baicalein-treated, and wogonoside-treated cells was markedly higher than that in the LPS-treated group. This indicated that the degradation of I*κ*B-*α* was inhibited by meranzin, baicalein, and wogonoside (Figures 10(a) and 10(c)). These findings suggest that meranzin, baicalin, and baicalein may inhibit the nuclear translocation of NF-*κ*B by inhibiting the degradation of I*κ*B-*α*. Baicalin, baicalein, and wogonoside inhibition of NF-*κ*B activation inhibits LPS-induced inflammatory factor production, which is consistent with results reported in previous studies [25–27].

## 4. Discussion

A single TCM contains hundreds of phytochemical components, while CMP is composed of two or more kinds of TCM. Thus, identification of the active components in these complex formulations is a major challenge. Recently, the spectrum-effect relationship has been used to identify the active components of TCM formulations. In this study, the anti-inflammatory components of QJHTT were screened using GRA, PLSR, and RDA. The findings of this study revealed that multiple components exhibited anti-inflammatory activities. Moreover, the different active components of CMP exhibited variable pharmacological activities. Peaks 11, 12, 13, 14, and 16 were strongly correlated with anti-inflammatory activity. This result was validated using all three spectrum-effect analysis methods. Comparing the results of GRA and PLSR of our research data with the published reference, as shown in Table S4, the consistency of the GRA and PLSR results in this study is better than in other similar published articles. In this study, both the GRA and PLSR results indicated that peaks 11, 12, 13, 14, and 16 were highly correlated with anti-inflammatory activity. However, in four of the other five published spectrum-effect relationship analysis articles, the GRA and PLSR results were not consistent. Therefore, multivariate statistical analysis is a reliable method for screening the active components of QJHTT. Peaks 11, 12, 13, 14, and 16 are potential anti-inflammatory components.

TCM formulations are natural combinations of many components. Screening of the active components in the TCM formulation can aid in identifying a new combination of drugs to improve the therapeutic efficacy of the formulation. Spectrum-effect relationship analysis can aid in screening a combination of drugs. Combination drugs have recently been proposed for the secondary development of TCM [28]. Based on the results of this study, we would further investigate the synergistic anti-inflammatory effects of the compounds meranzin, baicalin, baicalein, chrysin-7-O-beta-D-glucuronide, and wogonoside. Drug combinations can achieve better efficacy and fewer side effects due to the synergistic effect compared to single components [29]. Considering that combination drugs are composed of two or more drugs, the screening of their appropriate drug combinations is a great challenge [30]. This study provides a foundation for drug combinations of screened active ingredients. In contrast to the traditional method of spectrum-effect analysis, where different original herbs are collected for spectrum-effect analysis, the screened potential active ingredients are less accurate because of their high similarity and minor differences in composition between samples. In this study, extraction with different polar solvents yielded most of the components in the original herbs. This made the content of the components vary significantly between groups, which is more conducive to screening potential active ingredients with higher accuracy.

QJHTT is a classic CMP used to treat pulmonary inflammation and lung fever-induced cough. However, the active components of QJHTT have not been identified, which has significantly limited its clinical application. In this study, the anti-inflammatory bioactive components of QJHTT were identified using a multivariate statistical analysis. Among the five potential components with anti-inflammatory activity, meranzin exhibited anti-inflammatory activity in a xylene-induced ear-swelling mouse model [31]. Baicalin and baicalein have been reported to exhibit antioxidant and anti-inflammatory activities in diabetes, cardiovascular disease, and inflammatory bowel diseases by attenuating the activity of NF-*κ*B and inhibiting the secretion of several inflammatory cytokines [32]. Meranzin is a component of Citri Grandis Exocarpium, while baicalin, baicalein, and chrysin-7-O-*β*-D-glucuronide are components of Scutellariae Radix. Additionally, wogonoside is a component of Scutellariae Radix. Fritillariae Thunbrgii Bulbus, Scutellariae Radix, and Citri Grandis Exocarpium are used to treat lung fever-induced cough (induced by the inflammation of the lungs and bronchial tubes). The anti-inflammatory components predicted using spectrum-effect relationship analysis were experimentally validated *in vitro*. Meranzin, baicalin, baicalein, and wogonoside mitigated LPS-induced inflammation and enhanced the secretion of inflammatory factors, such as TNF-*α*, IL-4, and IL-6 in macrophages. Meranzin is the first reported inhibitor of LPS-induced inflammation in macrophages. Nuclear translocation of NF-*κ*B (p65) from the cytoplasm promotes the transcription and secretion of inflammatory factors by binding to the promoter region of their genes. The degradation of I*κ*B-*α*, an inhibitor of NF-*κ*B, activates the two subunits of NF-*κ*B, which translocate to the nucleus and initiate the expression of inflammatory factors [33]. These results indicated that meranzin, baicalein, and wogonoside inhibited the nuclear translocation of NF-*κ*B by inhibiting the degradation of I*κ*B-*α*. Although baicalin inhibited the nuclear translocation of NF-*κ*B, it did not markedly promote the degradation of I*κ*B-*α*. These results indicate the reliability of the potential active components obtained from the screening.

## 5. Conclusions

In this study, we successfully screened the active anti-inflammatory components of QJHTT using a multivariate statistical analysis method. The method involved analyzing the spectrum-effect relationship of different polar extracts of QJHTT using three correlation models. The active components (meranzin, baicalin, baicalein, chrysin-7-O-beta-D-glucuronide, and wogonoside) mediating the anti-inflammatory effects of QJHTT were identified. Meranzin, baicalin, baicalein, and wogonoside inhibited the nuclear translocation of NF-*κ*B and consequently inhibited the activation of inflammatory pathways. Further studies are needed to explore the differences in the efficacy of anti-inflammatory components of CMP.

## Figures and Tables

**Table 1:** Sources of the herbal materials of Qing Jin Hua Tan Tang.

Chinese name	Latin name	Lot number	Place of origin
Huangqin	Scutellariae Radix	180501541	Hebei
Zhizi	Gardeniae Fructus	180701741	Jiangxi
Jiegeng	Platycodonis Radix	180503213	Neimenggu
Maidong	Ophiopogonis Radix	180705081	Sichuan
Sangbaipi	Mori Cortex	180501871	Jiangsu
Zhimu	Anemarrhenae Rhizoma	171206831	Hebei
Gualouren	Trichosanthis Semen	170502031	Zhejiang
Huajuhong	Citri Grandis Exocarpium	180402681	Guangdong
Chuanbeimu	Fritillariae Cirrhosae Bulbus	180605411	Sichuan
Fuling	Poria	181002531	Hunan
Gancao	Glycyrrhizae Radix et Rhizoma	180703071	Neimenggu

**Table 2:** Anti-inflammatory activity of 10 Qing Jin Hua Tan Tang extracts.

	Model (%)	Dexamethasone acetate	WE-1	WE-2	EAWE-1	EAWE-2	NBWE-1
SIR	0	69.8 ± 8.03%	33.9 ± 11.9%	23.1 ± 5.60%	69.3 ± 7.31%	55.3 ± 5.27%	71.3 ± 2.73%
			NBWE-2	AE-1	AE-2	EAAE-1	EAAE-2
			62.5 ± 8.18%	74.3 ± 9.70%	48.6 ± 4.90%	34.8 ± 2.60%	27.6 ± 3.10%

^∗^WE-1, high-dose of water extract; WE-2, low-dose of water extract; EAWE-1, high-dose of ethyl acetate fraction of WE-1; EAWE-2, low-dose of ethyl acetate fraction of WE-2; NBWE-1, high-dose of *n*-butyl alcohol fraction of WE-1; NBWE-2, low-dose of *n*-butyl alcohol fraction of WE-2; AE-1, high-dose of alcohol extract; AE-2: low-dose of alcohol extract. Concentration of high dose of the extract: 400 mg/kg bodyweight/day; concentration of low dose of the extract: 200 mg/kg bodyweight/day.

**Table 3:** Coefficient of correlation of Qing Jin Hua Tan Tang components with their pharmacodynamics (swell inhibition rate (SIR)).

SIR	
Peak	Correlation coefficient
*P*13	0.795
*P*16	0.761
*P*14	0.760
*P*11	0.754
*P*12	0.735
*P*9	0.685
*P*6	0.682
*P*21	0.678
*P*7	0.671
*P*24	0.665
*P*19	0.659
*P*4	0.658
*P*17	0.657
*P*5	0.656
*P*1	0.655
*P*20	0.654
*P*8	0.654
*P*2	0.648
*P*18	0.643
*P*10	0.636
*P*22	0.621
*P*3	0.611
*P*15	0.585
*P*23	0.566

## Data Availability

The data used to support the results of this study are included within this article.

## Abbreviations

QJHTT: Qing Jin Hua Tan Tang
COPD: Chronic obstructive pulmonary disease
CMP: Chinese medicine prescription
TCM: Traditional Chinese medicine
LPS: Lipopolysaccharide
GRA: Gray relational analysis
PLSR: Partial least squares regression
RDA: Redundancy analysis
WE-1: High-dose water extract
WE-2: Low-dose water extract
EAWE-1: High-dose ethyl acetate fraction of WE-1
EAWE-2: Low-dose ethyl acetate fraction of WE-2
NBWE-1: High-dose *n*-butyl alcohol fraction of WE-1
NBWE-2: Low-dose *n*-butyl alcohol fraction of WE-2
AE-1: High-dose alcohol extract
AE-2: Low-dose alcohol extract
UHPLC: Ultrahigh-performance liquid chromatography
Q-TOF-MS/MS: Quadrupole time-of-flight tandem mass spectrometry
ESI: Electrospray ionization
CMC-Na: Sodium carboxymethylcellulose
SIR: Swell inhibition rate.

## Supplementary Materials

Supplementary Materials The preparation process of ten different polar extracts from QJHTT, the results of optimized detection wavelength, the product ion spectra of potentially active components, and the quantitative and qualitative results of potentially active components are exhibited in supplementary materials. .
