# Supplementary material for: Screening of Anti-Inflammatory Components of Qin Jin Hua Tan Tang by a Multivariate Statistical Analysis Approach for Spectrum-Effect Relationships
Source: J Anal Methods Chem. 2021 Aug 13;2021:6348979. doi: 10.1155/2021/6348979 (PMC8380171; doi:10.1155/2021/6348979)
Supplement: Supplementary Materials — The preparation process of ten different polar extracts from QJHTT, the results of optimized detection wavelength, the product ion spectra of potentially active components, and the quantitative and qualitative results of potentially active components are exhibited in supplementary materials. . [file 6348979.f1.docx]

**Supplementary materials**

**Screening of anti-inflammatory components of Qin Jin Hua Tan Tang by a multivariate statistical analysis approach for spectrum-effect relationships**

Feipeng Duan^1,2^, Yisheng Li^1^, Meizhen Zhao^1^, Tianyong Hu^1^, Xinquan Pan^1^, Yue Feng^1^, Fang Ma^1^, Shuqi Qiu^1*^, Yiqing Zheng^2*^

*^1^ Department of Otolaryngology，Longgang E.N.T hospital & Shenzhen Key Laboratory of E.N.T, Institute of E.N.T, Shenzhen, 518172, China*

*^2^ Institute of Otolaryngology, Institute of Hearing and Speech of Sun Yat-sen University, Sun Yat-sen Memorial Hospital, Sun Yat-sen University, Guangzhou, 510520, China*

**Running title: Screen the active sites of Qing Jin Hua Tan Tang**

***Correspondence author:** Yiqing Zheng, Institute of Otolaryngology,Institute of Hearing and Speech of Sun Yat-sen University, Sun Yat-sen Memorial Hospital, Sun Yat-sen University, Guangzhou, 510520, China. Email: zhengyiq@mail.sysu.edu.cn;

Shuqi Qiu, Department of Otolaryngology，Longgang E.N.T hospital & Shenzhen Key Laboratory of E.N.T, Institute of E.N.T, Shenzhen, 518172, China. Email:

Email: qiuqi66858@163.com;

1. **Extraction procedure of ten different polar extracts from QJHTT**

1.1 Water extracted group

Based on the composition of the QJHTT prescription，Scutellariae Radix (HQ) 55.8 g, Gardeniae Fructus (ZZ) 55.8 g, Platycodonis Radix (JG) 74.2 g, Ophiopogonis Radix (MD) 37.2 g, Mori Cortex (SBP) 37.2 g, Anemarrhenae Rhizoma (ZM) 37.2 g, Trichosanthis Semen (GLR) 37.2 g, Citri Grandis Exocarpium (HJH) 37.2 g, [Fritillariae Thunbrgii Bulbus](https://tcmspw.com/tcmspsearch.php?qr=Fritillariae%20Thunbrgii%20Bulbus&qsr=herb_en_name&token=c915820261f5a51a281b57995b782d9d) (ZBM) 37.2 g, Poria (FL) 37.2 g, and Glycyrrhizae Radix Et Rhizoma (GC) 7.44 g, with eight times the amount of water soak for two hours, extract for 1h, and filter the residue. Residues continued extracting for 1h with six times the amount of water, combined with the decoction and concentrated to a 600 mL volume. 600 mL is divided into three parts, one part in 200 mL, with reduced pressure to recover solvent to obtain a water extract (WE), the other two parts of 100 mL extracts were extracted with ethyl acetate (300 mL) and n-butanol (300 mL) respectively, collecting the ethyl acetate and n-butanol layer, repeat three times. Subsequently, combined the ethyl acetate and n-butanol layer of three times extracting respectively, concentrated under reduced pressure, so obtained the ethyl acetate extracts part from WE (EAWE), n-butyl alcohol extracts part from WE (NBWE). 1.2 Ethanol extracted group

Based on the composition of the QJHTT prescription，Scutellariae Radix (HQ) 55.8 g, Gardeniae Fructus (ZZ) 55.8 g, Platycodonis Radix (JG) 74.2 g, Ophiopogonis Radix (MD) 37.2 g, Mori Cortex (SBP) 37.2 g, Anemarrhenae Rhizoma (ZM) 37.2 g, Trichosanthis Semen (GLR) 37.2 g, Citri Grandis Exocarpium (HJH) 37.2 g, [Fritillariae Thunbrgii Bulbus](https://tcmspw.com/tcmspsearch.php?qr=Fritillariae%20Thunbrgii%20Bulbus&qsr=herb_en_name&token=c915820261f5a51a281b57995b782d9d) (ZBM) 37.2 g, Poria (FL) 37.2 g, and Glycyrrhizae Radix Et Rhizoma (GC) 7.44 g, with eight times the amount of 90% ethanol soak for two hours, extract for 1h, and filter the residue. Residues continued extracting 90% ethanol for 1h with six times the amount of water, combined with the decoction and concentrated to a 600 mL volume. Divide 600 mL into two parts, one of 400 mL and one of 200 mL, 400 mL concentrated decoction with reduced pressure to recover solvent from obtaining an ethanol extract (AE). The other parts of 200 mL extracts were extracted with ethyl acetate (300 mL), collecting the ethyl acetate layer, repeat three times. Subsequently, combined the ethyl acetate layer of three times extracting, concentrated under reduced pressure, so obtained the ethyl acetate extracts part from AE (EAAE).

**2. The comparsion of the chromatograms of QJHTT in different wavelengths**


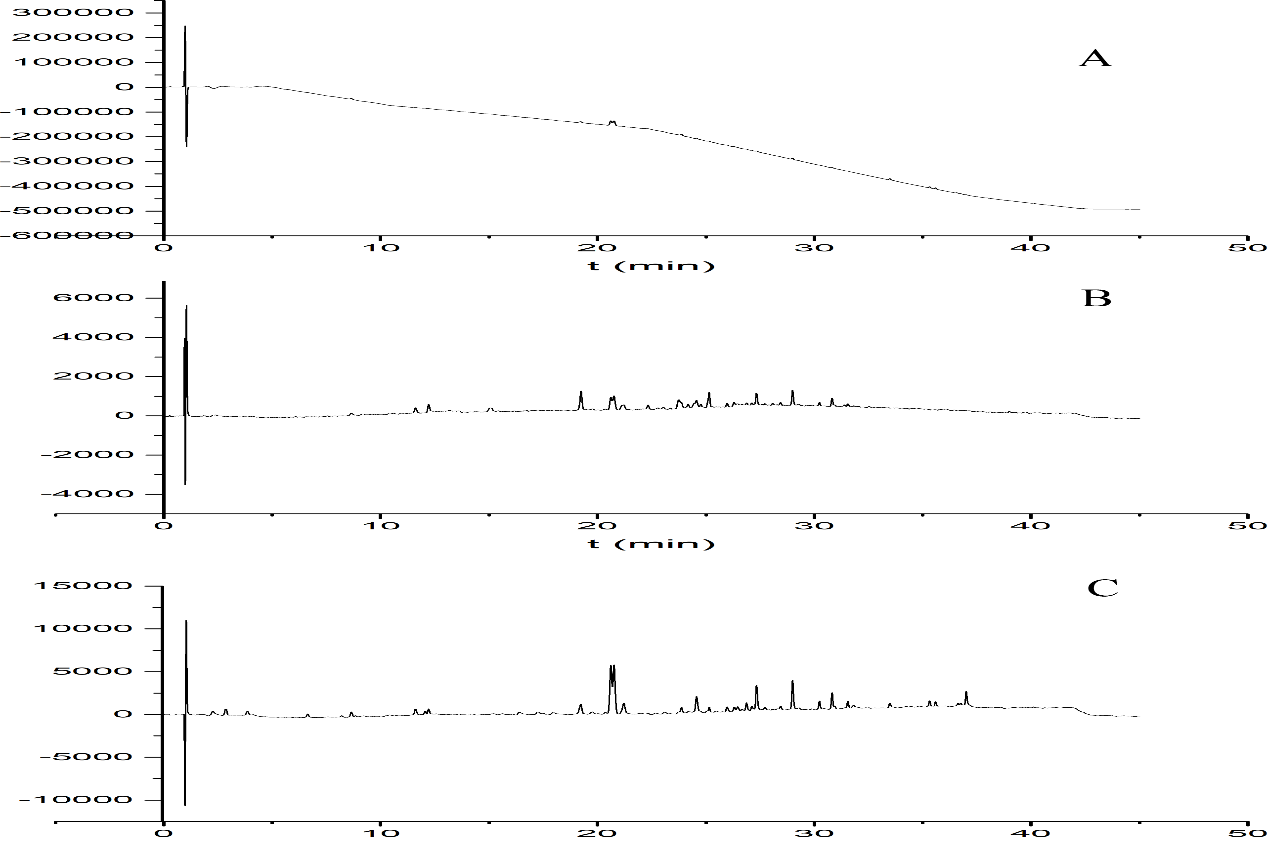


**Fig. S1** Different wavelengths chromatograph of NBWE-1: A: Detcetion wavelength at 210 nm; B: Detcetion wavelength at 266 nm; C: Detcetion wavelength at 360 nm.

**3.The product ion spectra of meranzin, baicalin, baicalein, chrysin-7-O-Beta-D-glucuronide, and wogonoside**

**
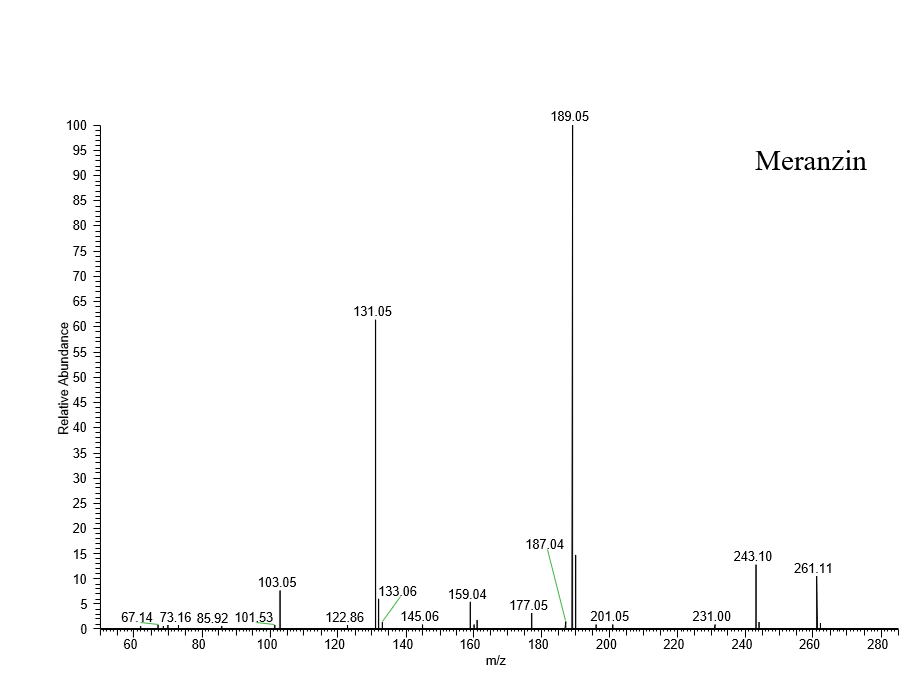
**

**Fig. S2** The product ion spectra of Meranzin


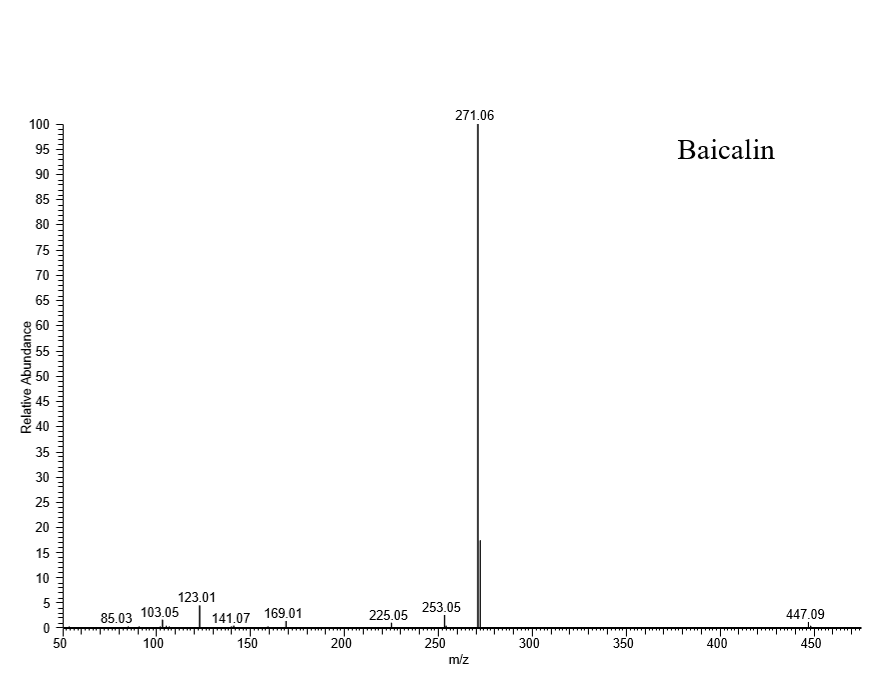


**Fig. S3** The product ion spectra of Baicalin.


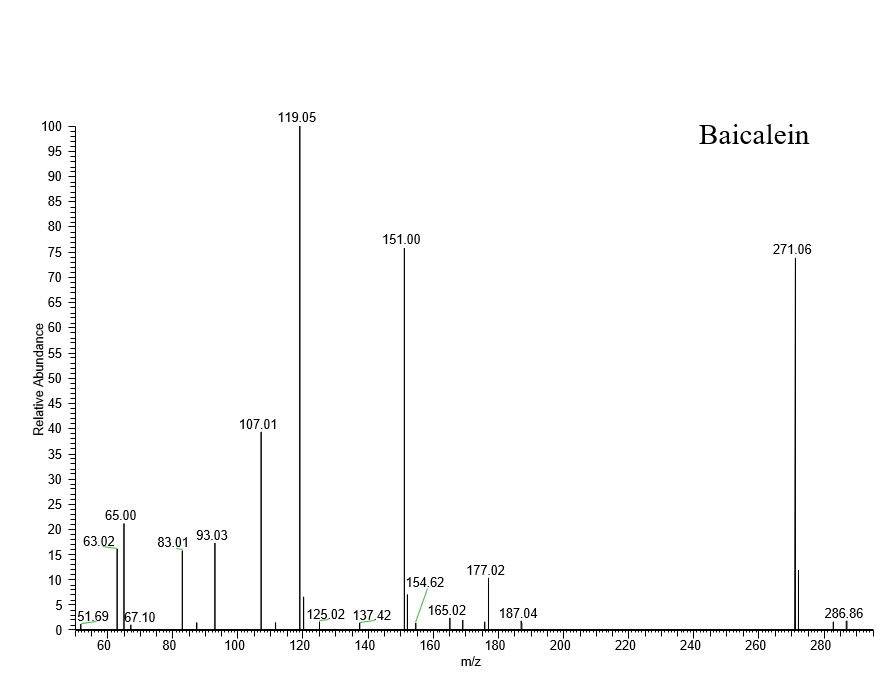


**Fig. S4** The product ion spectra of Baicalein.

**
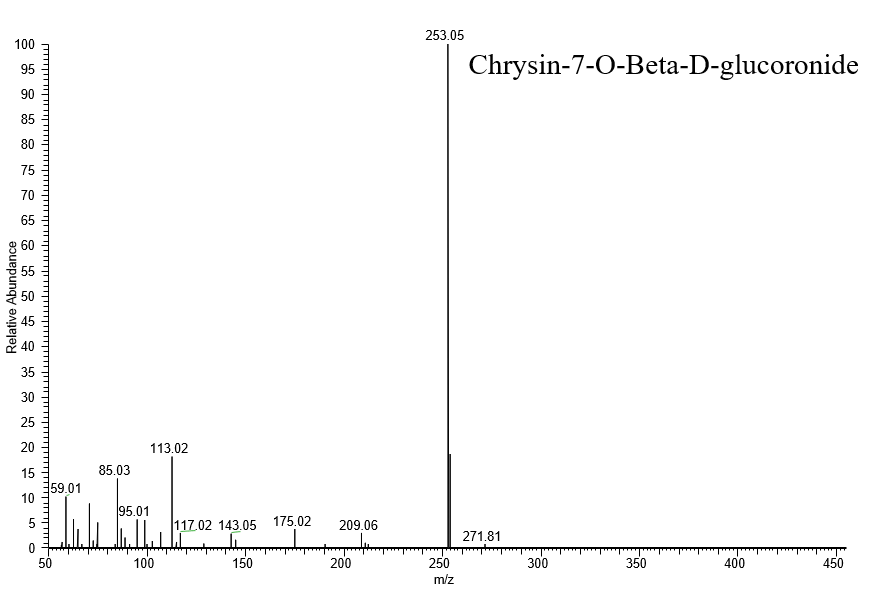
**

**Fig. S5** The product ion spectra of Chrysin-7-O-Beta-D-glucoronide.


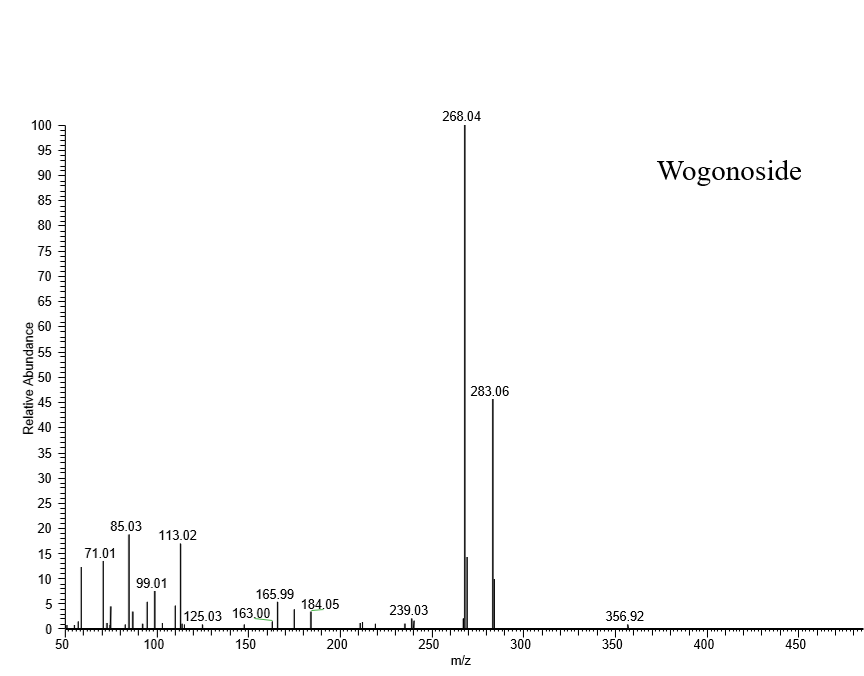


**Fig. S6** The product ion spectra of Wogonoside.

**Table S1** There were 24 peaks in the chromatograms of 10 batches of QJHTT extracts.

|  |  | Peak area | | | | | | | | | |
| --- | --- | --- | --- | --- | --- | --- | --- | --- | --- | --- | --- |
| Peak number | T(min) | WE-1 | WE-2 | EAWE-1 | EAWE-2 | NBWE-1 | NBWE-2 | AE-1 | AE-2 | EAAE-1 | EAAE-2 |
| Peak1 | 2.86 | 0.0 | 0.0 | 0.0 | 0.0 | 5675.9 | 3280.7 | 907.1 | 556.4 | 0.0 | 0.0 |
| Peak2 | 3.87 | 0.0 | 0.0 | 0.0 | 0.0 | 4387.6 | 2536.1 | 513.4 | 314.9 | 0.0 | 0.0 |
| Peak3 | 6.64 | 1484.4 | 854.7 | 0.0 | 0.0 | 2823.9 | 1632.2 | 449.0 | 275.4 | 0.0 | 0.0 |
| Peak4 | 8.65 | 0.0 | 0.0 | 0.0 | 0.0 | 3729.8 | 2155.8 | 673.3 | 413.0 | 0.0 | 0.0 |
| Peak5 | 11.61 | 0.0 | 0.0 | 0.0 | 0.0 | 5413.6 | 3129.1 | 919.9 | 564.2 | 0.0 | 0.0 |
| Peak6 | 12.12 | 1730.5 | 1013.0 | 2498.5 | 1856.8 | 2388.9 | 1380.8 | 5886.7 | 3610.9 | 4145.6 | 2591.4 |
| Peak7 | 12.21 | 3323.6 | 1937.8 | 3695.0 | 2646.1 | 3638.9 | 2103.3 | 6479.5 | 3974.5 | 5256.6 | 3296.4 |
| Peak8 | 19.22 | 0.0 | 0.0 | 0.0 | 0.0 | 5680.8 | 3283.5 | 2731.2 | 1675.3 | 3178.8 | 2200.1 |
| Peak9 | 20.75 | 2798.4 | 1750.0 | 0.0 | 0.0 | 40731.8 | 23543.0 | 19703.1 | 12085.9 | 6279.3 | 3988.0 |
| Peak10 | 21.2 | 0.0 | 0.0 | 0.0 | 0.0 | 11439.5 | 6612.1 | 1495.2 | 917.2 | 1364.6 | 907.6 |
| Peak11 | 23.86 | 0.0 | 0.0 | 2127.6 | 1581.2 | 6206.4 | 3587.3 | 2780.4 | 1705.5 | 0.0 | 0.0 |
| Peak12 | 24.56 | 26428.4 | 16998.7 | 45870.6 | 34091.0 | 12943.0 | 7481.0 | 61866.2 | 37948.7 | 25217.5 | 16267.8 |
| Peak13 | 25.97 | 1943.3 | 1249.9 | 3804.8 | 2827.7 | 4151.5 | 2399.6 | 5110.2 | 3134.6 | 3255.5 | 2100.1 |
| Peak14 | 26.45 | 1713.6 | 1102.2 | 2753.0 | 2046.0 | 3397.5 | 1963.8 | 3494.9 | 2143.8 | 2863.3 | 1847.1 |
| Peak15 | 26.87 | 40079.1 | 25778.9 | 7370.0 | 5477.4 | 5865.7 | 3390.4 | 10346.4 | 6346.5 | 6178.9 | 3986.0 |
| Peak16 | 27.33 | 8412.1 | 5410.6 | 16216.5 | 12052.1 | 17745.6 | 10257.0 | 23359.9 | 14329.0 | 15903.3 | 10259.2 |
| Peak17 | 28.99 | 0.0 | 0.0 | 2608.5 | 1938.6 | 18217.7 | 10529.8 | 5329.5 | 3269.1 | 5623.0 | 3627.4 |
| Peak18 | 30.23 | 0.0 | 0.0 | 1172.3 | 871.2 | 4852.0 | 2804.5 | 1307.1 | 801.8 | 2470.5 | 1593.7 |
| Peak19 | 30.81 | 0.0 | 0.0 | 1883.3 | 1399.6 | 10038.7 | 5802.3 | 3225.3 | 1978.4 | 3566.6 | 2300.8 |
| Peak20 | 31.54 | 0.0 | 0.0 | 1494.5 | 1110.7 | 4425.9 | 2558.2 | 861.9 | 528.7 | 1959.2 | 1263.9 |
| Peak21 | 33.48 | 3275.6 | 2106.9 | 3847.9 | 2859.8 | 3125.4 | 1806.5 | 3674.7 | 2254.1 | 3977.7 | 2566.0 |
| Peak22 | 35.32 | 4465.4 | 2872.2 | 3678.5 | 2733.9 | 3013.5 | 1741.8 | 3175.9 | 1948.1 | 3367.0 | 2172.1 |
| Peak23 | 35.59 | 3689.2 | 2372.9 | 2054.7 | 1527.0 | 2108.6 | 1218.8 | 2153.0 | 1320.7 | 2356.3 | 1520.0 |
| Peak24 | 37.01 | 15423.4 | 9920.3 | 12689.3 | 9430.7 | 13604.1 | 7863.2 | 14531.0 | 8913.3 | 13481.5 | 8696.9 |

**Table S2** The identification of Peaks 11, 12, 13, 14, and 16.

| No. | t_R_  (min) | select ion | Formula | measured mass | calculated mass | Error  (ppm) | MS/MS fragmentation | Identification |
| --- | --- | --- | --- | --- | --- | --- | --- | --- |
| 11 | 23.86 | [M+H]^+^ | C15H16O4 | 261.1128 | 261.1134 | -2.44 | 243.05,189.05 | Meranzin^[1,2]^ |
| 12 | 24.56 | [M+H]^+^ | C21H18O11 | 447.0901 | 447.0922 | -4.84 | 271.06 | Baicalin^[3]^ |
| 13 | 26.07 | [M+H]^+^ | C15H10O5 | 271.0620 | 271.0611 | -1.54 | 177.02, 151.00 | Baicalein^[3]^ |
| 14 | 26.45 | [M-H]^-^ | C21H18O10 | 429.0833 | 429.0827 | -1.39 | 253.05,175.00 | Chrysin-7-O-Beta-D-glucoronide^[4]^ |
| 16 | 27.33 | [M-H]^-^ | C22H20O11 | 459.0938 | 459.0933 | -1.24 | 356.92,283.06 | wogonoside^[3]^ |

**Table S3** The content of Peaks 11, 12, 13, and 16 in 10 extracts.

| µg/mL | WE-1 | WE-2 | EAWE-1 | EAWE-2 | NBWE-1 | NBWE-2 | AE-1 | AE-2 | EAAE-1 | EAAE-2 |
| --- | --- | --- | --- | --- | --- | --- | --- | --- | --- | --- |
| meranzin | 0 | 0 | 0.55 | 0.23 | 1.4 | 0.75 | 0.69 | 0.36 | 0 | 0 |
| baicalin | 48.44 | 23.32 | 83.75 | 42.36 | 23.96 | 12.04 | 112.79 | 59.36 | 46.24 | 23.99 |
| baicalein | 0.99 | 0.42 | 1.15 | 0.66 | 1.18 | 0.63 | 1.26 | 0.59 | 1.1 | 0.52 |
| wogonoside | 9.49 | 4.74 | 18.21 | 9.56 | 19.92 | 9.55 | 26.19 | 14.1 | 17.86 | 8.56 |

**Table S4** Comparing the results of GRA and PLSR of our research to published data.

| References | GRA | PLSR | Activities |
| --- | --- | --- | --- |
| Sai Gao, et al ^[5]^. | 4, 25, 26, 27, 28 | 4, 7, 11, 23, 17 | xanthine oxidase inhibitory |
| Xin Qiao, et al ^[6]^. | 27, 30, 31, 33,34 | 27, 30, 31, 33,34 | hemostasis |
| Yinrui Chen, et al ^[7]^. | 6, 7, 8, 9, 11 | 6, 7, 8, 13, 17 | anti-inflammatory |
| Jidan Zhang, et al ^[8]^. | 29, 31, 32, 33, 34 | 30, 35, 36, 37,39 | vasorelaxant |
| Zhengmeng Jiang, et al ^[9]^. | 1, 3, 11, 13, 14 | 3, 4, 6, 13, 14 | anti-inflammatory |
| This paper | 11,12, 13,14, 16 | 11,12,13,14,16 | anti-inflammatory |

**References**

[1] Q. D. Liu, C. Y. Xie, L. L. Yan, X. J. Xu and D. P. Yang, “High Performance Liquid Chromatography-DAD-Mass Spectrometry Analysis of Citri Grandis Exocarpium,” *World Science and Technology/Modernization of Traditional Chinese Medicine and Materia Medica*, vol. 13, pp. 864-867, 2011.

[2] G. J. Li, H. J. Wu, Y. Wang, W. L. Hung and R. L. Rouseff, “Determination of citrus juice coumarins, furanocoumarins and methoxylated flavones using solid phase extraction and HPLC with photodiode array and fluorescence detection,” *Food Chem*, vol. 271, pp. 29-38, 2019.

[3] J. Han, M. Ye, M. Xu, J. H. Sun, B. R. Wang and D. Guo, “Characterization of flavonoids in the traditional Chinese herbal medicine-Huangqin by liquid chromatography coupled with electrospray ionization mass spectrometry,” *Journal of Chromatography B*, vol. 848, pp. 355-362, 2007.

[4] L. Zhang, R. W. Zhang, Q. Li, W et al, “Development of the fingerprints for the quality evaluation of Seutellariae Radix by HPLC-DAD and LC-MS-MS,” *Chromatographia*, vol. 66, pp.13-20, 2007.

[5] S. Gao, H. Chen, and X. Zhou, “Study on the spectrum-effect relationship of the xanthine oxidase inhibitory activity of Ligustrum lucidum,” *J Sep Sci*, vol. 42, no. 21, pp. 3281-3292, 2019.

[6] X. Qiao, C. Qu, Q. Luo, et al, “UHPLC-qMS spectrum-effect relationships for Rhizoma Paridis extracts,” *J Pharm Biomed Anal*, vol. 194, Article ID 113770, 2021.

[7] Y. Chen, S. Zou, W. Xu, Q. Sun, and L. Yun, “Spectrum-effect relationship of antioxidant and anti-inflammatory activities of Laportea bulbifera based on multivariate statistical analysis,” *Biomed Chromatogr*, vol. 34, no. 2, Article ID e4734, 2020.

[8] J. D. Zhang, T. Chen, K. Li, et al. “Screening active ingredients of rosemary based on spectrum-effect relationships between UPLC fingerprint and vasorelaxant activity using three chemometrics,” *J Chromatogr B Analyt Technol Biomed Life Sci*, vol.15, pp. 1134-1135. 2019.

[9] Z. M. Jiang, C. Zhang, X. J. Gong, et al. “Quantification and efficient discovery of quality control markers for Emilia prenanthoidea DC. by Fingerprint-Efficacy Relationship Modelling,” *J Pharm Biomed Anal*, vol. 156, pp. 36-44, 2018.
